# T-cell Large Granular Lymphocytic Leukemia and Felty Syndrome in Rheumatoid Arthritis: A Case Report

**DOI:** 10.7759/cureus.41780

**Published:** 2023-07-12

**Authors:** Supritha Prasad, Iman Mushfiq Farooqui, Lujain AlZoubi, Shiva Arami

**Affiliations:** 1 Internal Medicine, University of Illinois at Chicago, Chicago, USA; 2 Medicine, Aga Khan University, Karachi, PAK; 3 Rheumatology, University of Illinois at Chicago, Chicago, USA; 4 Medicine, The University of Jordan, Amman, JOR

**Keywords:** large granular lymphocytic leukemia, rheumatoid arthritis, neutropenia, t-cell leukemia, felty’s syndrome, lgl leukemia

## Abstract

T-cell large granular lymphocytic (LGL) leukemia is characterized by a clonal proliferation of CD3+ T-cells and has been associated with rheumatoid arthritis (RA). Splenomegaly is a common finding and a majority of cases present with cytopenia. Felty syndrome (FS) is characterized by neutropenia and splenomegaly and is also classically described in the literature for its association with RA. Similarities in clinical features, pathogenesis, management, genetics, and immunologic basis of FS and T-cell LGL leukemia have led to the suggestion that they exist on the same spectrum of disease. We present a case of T-cell LGL leukemia in an RA patient with clinical features not distinguishable from features of FS.

## Introduction

Large granular lymphocytic (LGL) leukemia is a myeloproliferative disorder characterized by a clonal proliferation of CD3+ T-cells or CD3- natural killer (NK) cells [1]. About 11-36% of patients with T-cell LGL leukemia have rheumatoid arthritis (RA). In contrast, the prevalence of RA in the general adult worldwide population is less than 1% [2-4]. This suggests a strong association between T-cell LGL leukemia and RA. Felty syndrome (FS) is another extra-articular phenomenon that has been associated with RA. FS is described as a triad of neutropenia, splenomegaly, and concurrent RA. The current estimate of the incidence of FS in the RA population is 1-3% [5]. In addition to a strong association with RA, there are several suggested parallels in the clinical presentation, pathogenesis, treatment, and genetic and immunologic basis of both FS and T-cell LGL leukemia, and it has thus been suggested that they exist on the same spectrum of disease [6]. We present a case of T-cell LGL leukemia in an RA patient with clinical features indistinguishable from features of FS, supporting the idea FS and T-cell LGL lie on the same disease spectrum.

## Case presentation

Our patient is a 69-year-old male seen in the rheumatology clinic for routine follow-up. He first presented to the clinic in 2015 as a referral from an outside facility for RA management. His other chronic medical conditions upon presentation included type II diabetes mellitus managed with metformin and glipizide as well as hypothyroidism managed with levothyroxine. He reported no known family history of autoimmune disorders. Available information at the time suggested the patient was diagnosed with RA in 1999 and had previously been on methotrexate, leflunomide, and etanercept before being lost to follow-up due to issues with insurance. He therefore initially presented solely on prednisone. The physical exam was significant for active synovitis of the metacarpophalangeal (MCP) joints, multiple proximal interphalangeal (PIP) joints, and wrists. Repeat serologic testing in the clinic demonstrated an antinuclear antibody titer of 1:640, rheumatoid factor (RF) of 60 IU/mL (reference range = 0-14 IU/mL), and cyclic citrullinated peptide antibody (anti-CCP) value of 143 units (reference range = 0-19 units). Blood counts were within normal limits, although platelets were borderline low-normal at 175 (Table 1). X-ray imaging of bilateral hands and feet was without erosions. He was re-started on methotrexate weekly with folic acid with the continuation of prednisone daily. Approximately one month later, after insurance approval, he was re-initiated on etanercept.

**Table 1:** Patient’s complete blood count with differential upon initial presentation to the clinic and at the onset of leukopenia WBC: white blood cells; RBC: red blood cells; MCV: mean corpuscular volume; MCH: mean corpuscular hemoglobin; MCHC: mean corpuscular hemoglobin concentration; RDW: red blood cell distribution width; MPV: mean platelet volume.

Component	Reference range	Result: March 2015	Result: September 2017
WBC	3.9-12.0 K/UL	8.4 K/UL	2.9 K/UL
RBC	4.00-6.10 M/UL	4.84 M/UL	4.51 M/UL
Hemoglobin	13.2-18.0 GM/DL	14.6 GM/DL	13.9 GM/DL
Hematocrit	38.0-55.0%	44.9%	40.7%
MCV	80.0-99.0 FL	92.8 FL	90.4 FL
MCH	26.0-35.0 PG	30.1 PG	30.9 PG
MCHC	32.0-37.0 GM/DL	32.5 GM/DL	34.2 GM/DL
RDW	11.6-15.0%	12.6%	15.0%
Platelets	150-450 K/UL	175 K/UL	113 K/UL
MPV	6.5-11.0 FL	7.1 FL	7.8 FL
Neutrophils relative	40-70%	72.1%	25.3%
Neutrophils absolute	1.3-7.5 K/UL	6.1 K/UL	0.7 K/UL
Lymphocytes absolute	1.3-4.2 K/UL	1.7 K/UL	1.4 K/UL
Monocytes absolute	0.2-1.0 K/UL	0.5 K/UL	0.6 K/UL
Eosinophils absolute	0.0-0.5 K/UL	0.1 K/UL	0.1 K/UL
Basophils absolute	<=0.2 K/UL	0.1 K/UL	0.0 K/UL
Basophils relative	0.0-2.0%	0.9%	0.8%
Eosinophils relative	0-6%	1.4%	5.0%
Lymphocytes relative	25-45%	20.0%	49.3%
Monocytes relative	2-12%	5.6%	19.6%

Methotrexate was titrated and prednisone was tapered over several visits. Therapeutic drug monitoring during this time showed slight abnormalities in liver function tests, namely, slight elevations in aminotransferases, as well as borderline and intermittent thrombocytopenia. The additional investigation demonstrated no significant alcohol use and negative hepatitis panel. Given the elevated body mass index, weight loss was recommended with suspicion of non-alcoholic fatty liver disease.

**Figure 1:**
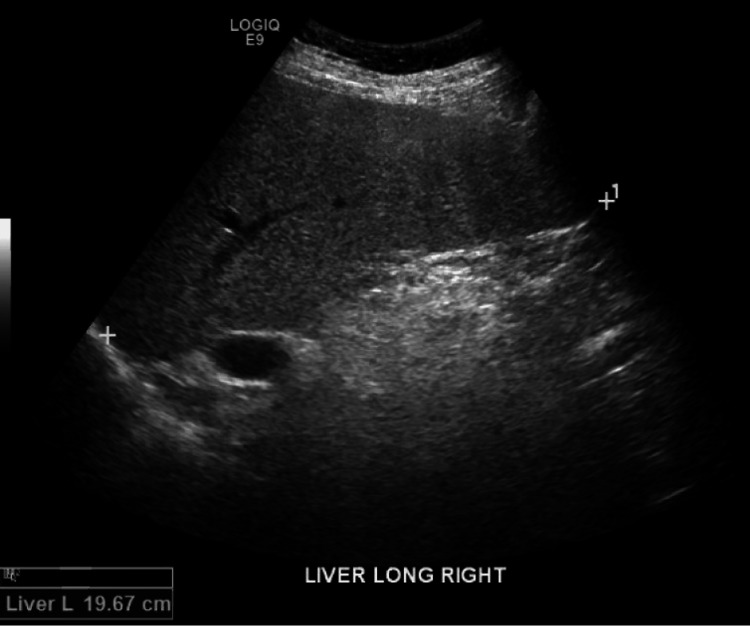
Hepatomegaly found on patient’s abdominal ultrasound

**Figure 2:**
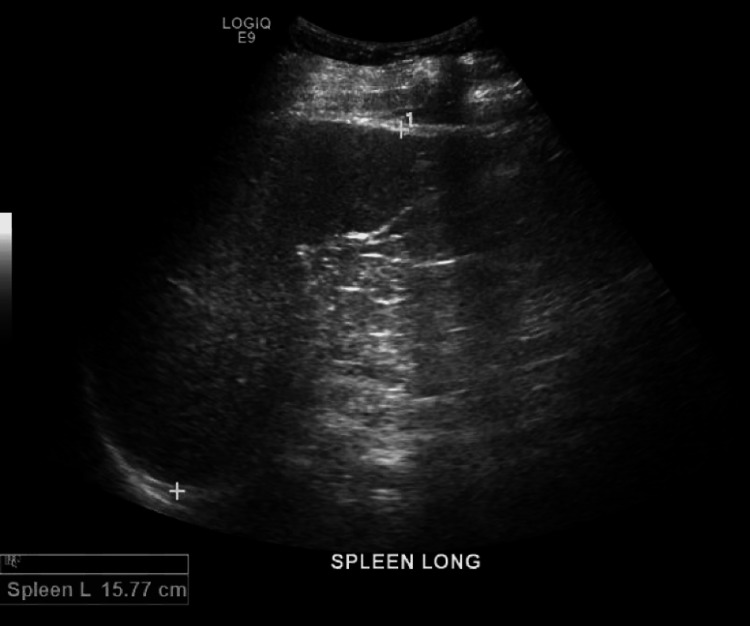
Splenomegaly found on patient’s abdominal ultrasound

In 2017, approximately two years after the initial presentation, routine lab tests demonstrated neutropenia in addition to worsening thrombocytopenia (Table 1), although the aminotransferase elevations had resolved. Methotrexate was discontinued. The neutropenia and thrombocytopenia persisted even in the absence of methotrexate. The patient was sent to the hematology clinic for further evaluation, given the concern for FS or LGL. Immunophenotyping on a peripheral blood sample raised suspicion for T-cell large granular lymphoproliferative disorder versus a reactive process (Table 2). Clonal T-cell receptor (TCR) was detected by polymerase chain reaction (PCR), consistent with the presence of a clonal T-cell population. A pathologist's review of a peripheral blood smear demonstrated neutropenia with large granular lymphocytes increased in number, representing approximately 35% of white blood cells. Ultrasound of the abdomen was significant for hepatomegaly with echotexture changes suggestive of hepatocellular disease or fatty infiltration, as well as splenomegaly (Figures 1, 2). Given an absolute neutrophil count (ANC) decreasing to 100, etanercept was discontinued.

**Table 2:** Results of immunophenotyping on patient’s peripheral blood sample GC: germinal center; NK: natural killer; PMNs: polymorphonuclear neutrophils; TCR: T-cell receptor. Interpretation: P = positive test; N = negative test; I = indeterminate test result; N.I. = test result not interpretable; ND = not done; SC = see comment. Intensity: Refers to the strength of antigen expression compared to normal cell counterpart. NL = normal, intensity = normal cell counterpart; D = Dim, intensity expression overlaps and is slightly greater than of normal cells; M = moderate, expression 1.0-1.5 logs greater; B = bright/strong, expression >1.5 logs greater; C = complex, cells of interest show spectrum or mixed intensity expressions; I = indeterminate; SC = see comment. Comment by interpreting physician: "The analysis demonstrates a subpopulation of NK-like T-cells, expressing dim CD2, CD3, dim CD5, CD57, and surface T-cell receptor alpha/beta, with decreased CD7 expression. This population represents approximately 27% of total cellular events (54% of gated T-cells) and consists of more CD8-positive cells than CD4-positive cells. This population is negative for CD10, CD16, CD25, CD30, and CD56 expression. In view of peripheral blood morphology, this population most likely represents T-cell large granular lymphocytes. The remaining T-cells express all pan-T-cell antigens analyzed, including CD2, CD3, CD5, and CD7. The CD4:CD8 ratio is reversed at 0.4:1. These immunophenotypic findings can be seen in large granular lymphocyte lymphoproliferative disorder as well as reactive conditions. A reactive process is favored. Recommend correlation with clinical history and other laboratory studies, including molecular studies for T-cell gene rearrangement."

Marker	Interpretation	Intensity
CD45	P	M
CD19 (Pan B-cell)	P	M
CD19/CD5 (B-cell subset)	N	
CD19/CD10 (Immature & GC B-cells)	N	
CD19/CD38 (Immature & GC B-cells)	P	D
CD20 (Maturing/mature B-cell)	P	M
CD23 (Mature activated B-cells; dendritic cells	P	D
CD138 (Plasma cells)	N	
CD19/sIgk (Surface Igk)	P	M
CD19/sIg 1 (Surface Ig1)	P	M
CD3 (Immature/mature T-cell)	P	M
CD3/TCR A/B (T-cell subset)	P	M
CD3/TCR G/D (T-cell subset)	P	D
CD3/CD4 (Helper T-cell)	P	M
CD3/CD8 (Cytotoxic/suppressor T-cell)	P	M
CD3/CD2 (Pan T-cell)	P	SC
CD3/CD5 (Pan T-cell)	P	SC
CD3/CD7 (Pan T-cell, NK cell)	P	SC
CD3/CD7- (T-cell subset)	P	SC
CD3/CD56 (T-cell subset)	N	
CD16 (NK-/cytotoxic T-cells; PMNs)	N	
CD3/CD57 (T-cell subset)	P	SC
CD3/CD25 (T-cell subset)	N	
CD3/CD30 (T-cell subset)	N	
CD3/CD4/CD10 (GC T-cell subset)	N	
CD3-/CD56 (NK cells)	P	M

Methotrexate was re-initiated at 12.5 mg weekly and titrated to the treatment dose for LGL at 10 mg/m^2^, equating to 25 mg weekly for our patient. Prednisone was increased to 60 mg daily for four weeks, followed by a stepwise taper of 10 mg every two weeks. Per chart documentation, this treatment regimen was guided by a phase II study by the Eastern Cooperative Oncology Group. A bone marrow biopsy was obtained to rule out other myeloproliferative processes. Biopsy analysis showed marrow with increased interstitial T-cells suggestive of LGL and the STAT3 mutation was detected. Our patient was maintained on treatment dose methotrexate and a high dose prednisone taper as well as prophylactic antimicrobials while the ANC was <1000. An increase in ANC was seen within four weeks; however, our patient continued to have fluctuations in white blood cell counts for several months, indicative of partial treatment response. It was thus recommended that the patient continue methotrexate for at least one year. Given the concern for active synovitis in the interim, he was started on hydroxychloroquine. He was also started on sulfasalazine but did not tolerate this therapy due to gastrointestinal symptoms. The patient was deemed to be in complete remission (CR) ~14 months after methotrexate treatment initiation for LGL with ANC > 1500. This finding, along with hemoglobin > 11 and platelet count > 100k, was maintained for at least one year, at which point methotrexate was tapered to 20 mg weekly (~8mg/m^2^), guided by control of our patient's RA symptoms.

## Discussion

T-cell LGL leukemia can present with neutropenia in about 70-80% of cases and splenomegaly in about 20-60% of cases. Both features overlap with the diagnostic criteria of FS [6-9]. It has been suggested that neutropenia in both diseases is also caused by similar mechanisms of survival and proliferation defects, such as dysregulated apoptosis, as opposed to defects in the production of neutrophils. It has also been hypothesized that both diseases arise due to the exposure of host T-cells to an exogenous trigger, likely a viral antigen [10-13]. Clonal expansion of CD3+, CD28-, and CD57+ T-cells has been noted in T-cell LGL leukemia as well as a subset of FS patients, and furthermore, both diseases have a similar prevalence of the human leukocyte antigen (HLA)-DR4 gene [6,14]. Since both T-cell LGL leukemia and FS have very similar features, it is not surprising that the treatment of both diseases is through immunosuppression, as was demonstrated via the utilization and success of methotrexate in our patient [15].

## Conclusions

Here, we have discussed a case of a patient with RA found to have T-cell LGL as well as a presentation consistent with FS. Through reporting this case, we aim to increase the identification of such diagnoses in our patients with RA. While cytopenia can commonly occur with the use of rheumatologic medications, we seek to highlight the importance of further evaluation of features like leukopenia when appropriate. We also aim to encourage collaboration between specialists in evaluating manifestations of such complex disease processes with the goal of optimizing treatment for our patients.

## References

[1] Sokol L, Loughran TP Jr (2006). Large granular lymphocyte leukemia. Oncologist.

[2] Moosic KB, Ananth K, Andrade F, Feith DJ, Darrah E, Loughran TP Jr (2022). Intersection between large granular lymphocyte leukemia and rheumatoid arthritis. Front Oncol.

[3] Loughran TP Jr (1993). Clonal diseases of large granular lymphocytes. Blood.

[4] Firestein GS (2003). Evolving concepts of rheumatoid arthritis. Nature.

[5] Owlia MB, Newman K, Akhtari M (2014). Felty's syndrome, insights and updates. Open Rheumatol J.

[6] Liu X, Loughran TP Jr (2011). The spectrum of large granular lymphocyte leukemia and Felty's syndrome. Curr Opin Hematol.

[7] Starkebaum G (2000). Leukemia of large granular lymphocytes and rheumatoid arthritis. Am J Med.

[8] Rose MG, Berliner N (2004). T-cell large granular lymphocyte leukemia and related disorders. Oncologist.

[9] Lamy T, Loughran TP Jr (2003). Clinical features of large granular lymphocyte leukemia. Semin Hematol.

[10] Breedveld FC, Lafeber GJ, de Vries E, van Krieken JH, Cats A (1986). Immune complexes and the pathogenesis of neutropenia in Felty's syndrome. Ann Rheum Dis.

[11] Melenhorst JJ, Brümmendorf TH, Kirby M, Lansdorp PM, Barrett AJ (2001). CD8+ T cells in large granular lymphocyte leukemia are not defective in activation- and replication-related apoptosis. Leuk Res.

[12] Loughran TP Jr, Sherman MP, Ruscetti FW (1994). Prototypical HTLV-I/II infection is rare in LGL leukemia. Leuk Res.

[13] Vallejo AN, Schirmer M, Weyand CM, Goronzy JJ (2000). Clonality and longevity of CD4+CD28null T cells are associated with defects in apoptotic pathways. J Immunol.

[14] Starkebaum G, Loughran TP Jr, Gaur LK, Davis P, Nepom BS (1997). Immunogenetic similarities between patients with Felty's syndrome and those with clonal expansions of large granular lymphocytes in rheumatoid arthritis. Arthritis Rheum.

[15] Lamy T, Moignet A, Loughran TP Jr (2017). LGL leukemia: from pathogenesis to treatment. Blood.

